# Influence of Homoepitaxial Layer Thickness on Flatness and Chemical Mechanical Planarization Induced Scratches of 4H-Silicon Carbide Epi-Wafers

**DOI:** 10.3390/mi16060710

**Published:** 2025-06-13

**Authors:** Chi-Hsiang Hsieh, Chiao-Yang Cheng, Yi-Kai Hsiao, Zi-Hao Wang, Chang-Ching Tu, Chao-Chang Arthur Chen, Po-Tsung Lee, Hao-Chung Kuo

**Affiliations:** 1 Department of Photonics, Institute of Electro-Optical Engineering, College of Electrical and Computer Engineering, National Yang Ming Chiao Tung University, Hsinchu 300093, Taiwan; rock.hsieh.ee11@nycu.edu.tw (C.-H.H.); potsung@nycu.edu.tw (P.-T.L.); 2 Wafer Technology Division, HuaHsu Silicon Materials Corporation, Taichung 407019, Taiwan; itri537366@itri.org.tw; 3 Semiconductor Research Center, Hon Hai Research Institute, Taipei 114699, Taiwan; jason.yk.hsiao@foxconn.com; 4 Academy of Innovative Semiconductor and Sustainable Manufacturing, National Cheng Kung University, Tainan 701401, Taiwan; zhwang@gs.ncku.edu.tw; 5 Department of Electrical Engineering, National Central University, Taoyuan 320317, Taiwan; 6 Department of Mechanical Engineering, National Taiwan University of Science and Technology, Taipei 106335, Taiwan; artchen@mail.ntust.edu.tw

**Keywords:** SiC thick epitaxial, SiC defect propagation, SiC wafer geometry

## Abstract

The integration of thick homoepitaxial layers on silicon carbide (SiC) substrates is critical for enabling high-voltage power devices, yet it remains challenged by substrate surface quality and wafer geometry evolution. This study investigates the relationship between substrate preparation—particularly chemical mechanical planarization (CMP)—and the impact on wafer bow, total thickness variation (TTV), local thickness variation (LTV), and defect propagation during epitaxial growth. Seven 150 mm, 4° off-axis, prime-grade 4H-SiC substrates from a single ingot were processed under high-volume manufacturing (HVM) conditions and grown with epitaxial layers ranging from 12 μm to 100 μm. Metrology revealed a strong correlation between increasing epitaxial thickness and geometric deformation, especially beyond 31 μm. Despite initial surface scratches from CMP, hydrogen etching and buffer layer deposition significantly mitigated scratch propagation, as confirmed through defect mapping and SEM/FIB analysis. These findings provide a deeper understanding of the substrate-to-epitaxy integration process and offer pathways to improve manufacturability and yield in thick-epilayer SiC device fabrication.

## 1. Introduction

Silicon carbide (SiC), particularly the 4H polytype, has emerged as a leading material for high-performance power electronics due to its wide bandgap (3.23 eV), high thermal conductivity, and exceptional breakdown electric field [1,2,3,4]. These characteristics make 4H-SiC especially suitable for next-generation high-voltage, high-temperature, and high-frequency devices [5].

Thicker epitaxial layers are especially desirable for high-voltage applications. For example, a 1 µm epitaxial layer on a SiC Schottky diode can support a breakdown voltage of approximately 118 V [6]. Consequently, to meet the stringent requirements of high-voltage device design, epitaxial layers typically need to be tens to hundreds of micrometers thick. To support such thick growth, the preparation of high-quality, low-defect substrates is essential. The chemical mechanical planarization (CMP) process is widely adopted in substrate fabrication to achieve the surface planarity and defect control required for epitaxy. However, CMP can introduce surface scratches and residual stress, which may impact the morphology and defect density of the subsequently grown epitaxial layers [7,8,9]. Wafer geometry characteristics, such as bow, warp, and total thickness variation (TTV), further complicate this integration, especially as epitaxial thickness increases [10,11,12].

While prior research has investigated individual aspects of wafer stress, geometry, and epitaxial defect formation, few studies have systematically correlated CMP-induced defects with wafer geometry evolution and defect transfer behavior across a range of epitaxial layer thicknesses under high-volume manufacturing (HVM) conditions. This is a critical gap, especially given industrial specifications that permit a finite level of scratches on SiC substrates based on wafer diameter. For instance, in 200 mm production, cumulative scratch lengths ≤50 mm are often tolerated—highlighting not only the practical limitations of eliminating such defects but also the need to understand how these residual surface imperfections interact with epitaxial processes and influence final wafer quality.

Recent advancements in epitaxial growth technologies, such as the incorporation of hydrogen (H_2_) pre-treatment and low-defect-density buffer layers, offer promising avenues for mitigating the propagation of CMP-induced substrate defects into the active epitaxial layer [13,14,15]. However, the quantitative effects of such mitigation strategies on wafer geometry, particularly under the influence of increasing epitaxial thickness, remain underexplored.

In this study, we examine seven 150 mm, 4° off-axis, prime-grade 4H-SiC substrates from a single ingot, processed with production-grade CMP and grown with homoepitaxial layers ranging from 12 μm to 100 μm using an Aixtron G10 warm-wall planetary CVD reactor under industry-representative conditions. Pre- and post-epitaxy metrology revealed that wafer bow, TTV, and LTV increase systematically with epitaxial thickness—particularly beyond 31 μm—while CMP-induced substrate scratches were effectively mitigated through H_2_ etching and buffer layer growth, showing minimal propagation into the epitaxial layers. These findings offer critical insight into substrate-to-epitaxy process integration and its impact on manufacturability and yield in thick-epilayer, high-voltage SiC device production.

## 2. Experimental Setup

Seven 150 mm-diameter, 350 µm-thick, (0001)-oriented Si-face 4° off-cut toward the [11,12,13,14,15,16,17,18,19,20] direction, N-type 4H-SiC substrates with a doping concentration of approximately 5 × 10^19^ cm^−3^ were selected from the tail section of the same SiC ingot. All substrates were of prime-grade quality and prepared on a high-volume manufacturing (HVM) line to ensure consistency.

### 2.1. Substrate Preparation

The epi-ready substrates were processed as follows:

1. Wafering: SiC ingots were sliced into 425 µm-thick wafers using a slurry-based multi-wire saw.
2. Edge Grinding: Performed to remove edge chipping and improve wafer handling.
3. Laser Marking: Each wafer was uniquely marked to ensure traceability and to associate each with a specific epitaxial condition.
4. Lapping: Conducted to flatten the wafers and remove saw damage.
5. Double-Side Grinding: Rough and fine grinding were carried out using #1200 and #8000 grinding wheels, respectively. The total thickness variation (TTV) was controlled within ±1 µm, and the surface roughness (Ra) after fine grinding was <3 nm.
6. Double-Side Chemical Mechanical Planarization (DSP): Executed in a Class 1000 environment using a commercial alkaline slurry with 150 nm mean-size Al_2_O_3_ abrasives and a polyurethane pad to remove 6 µm of material.
7. Single-Side Chemical Mechanical Planarization (SSP): Also performed in a Class 1000 environment using a colloidal silica (SiO_2_) slurry (100 nm mean abrasive size) and a suede pad, removing 1 µm on the Si-face. Final wafer thickness was 350 µm.
8. Post-CMP Cleaning: Wafers underwent sequential chemical cleaning using a tank-based system. The process included diluted hydrofluoric acid (dHF), followed by SC1 (NH_4_OH + H_2_O_2_ + DI water) and SC2 (HCl + H_2_O_2_ + DI water) cleans to eliminate organic and metallic contaminants. After chemical treatment, wafers were thoroughly rinsed with deionized water and dried in a Class 100 cleanroom environment to ensure surface cleanliness and minimize particulate contamination prior to epitaxial growth.
9. Defect Inspection: Conducted using a KLA Candela 8520 tool in a Class 10 mini-environment to map surface defects.
10. Substrate Geometry Measurement: Parameters such as bow, warp, TTV, and local thickness variation (LTV) were characterized using a CMIT (Cheng Mei Instrument Technology) GSS^+^ geometry inspection system with the Si-face facing upward, under Class 100 conditions.

Based on the mapped defect locations, scanning electron microscopy (SEM) and focused ion beam (FIB) techniques were employed for quantitative analysis of surface scratches.

### 2.2. Epitaxial Growth

**Figure 1:**
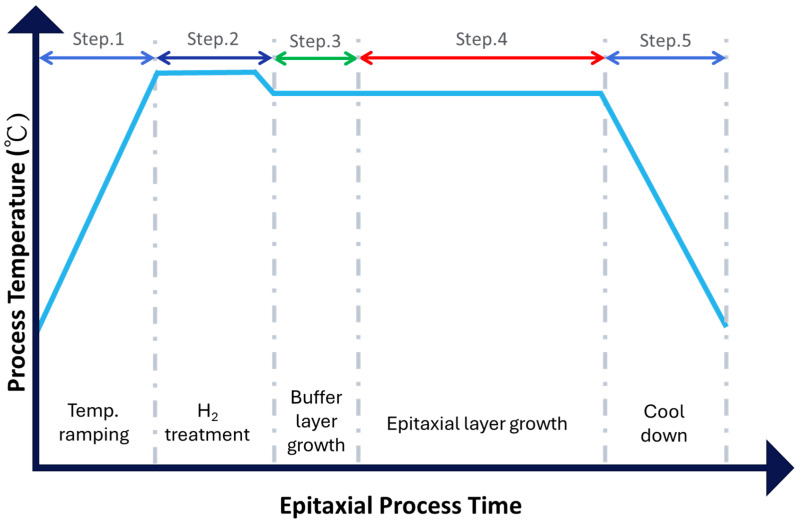
Temperature profile and purposes of each step in the epitaxial process.

Homoepitaxial SiC layers of varying thickness were deposited on the Si-face using an Aixtron G10 warm-wall planetary multi-wafer chemical vapor deposition (CVD) reactor. The epitaxial process, illustrated in Figure 1, consisted of the following steps:

1. Step 1 (Temperature Ramping): Reactor temperature increased from 600 ± 5 °C to 1630 ± 5 °C under a hydrogen (H_2_) and argon (Ar) atmosphere.
2. Step 2 (H_2_ Etching): A 15 min H_2_ pre-treatment (350 sccm) etched approximately 0.3 µm of material from the Si-face surface.
3. Step 3 (Buffer Layer Growth): A 1 µm-thick SiC buffer layer with a doping concentration of 1 × 10^18^ cm^−3^ was grown at 1600 ± 5 °C using SiHCl_3_ and C_2_H_4_ as precursors, with a C/Si ratio of 0.9, and a growth rate of 15 µm/h.
4. Step 4 (Main Epitaxial Growth): SiC epitaxial layers of 12 µm, 16 µm, 31 µm, 60 µm, and 100 µm thickness were grown with a doping concentration of 1 × 10^15^ cm^−3^. The growth rate was maintained at 20 µm/h with a C/Si ratio of 1.2 by adjusting the process time accordingly.
5. Step 5 (Cooling and Unloading): Upon completion of growth, wafers were cooled and unloaded from the reactor.

Epitaxial layer thickness uniformity across the seven wafers was maintained within 1.0–1.5%, as measured by Fourier Transform Infrared (FT-IR) spectrometry with a 3 mm edge exclusion. Post-growth, wafer geometry, and surface defect inspections were repeated using the same CMIT GSS^+^ and KLA Candela 8520 tools described in the substrate preparation phase.

## 3. Results and Discussions

### 3.1. Wafer Flatness Behavior with Epi Layer Thickness

According to the ASTM F534 wafer bow measurement definition, a higher positive bow indicates a greater +Z distance between the wafer surface and the best-fit plane at the wafer center when the wafer is unclamped, and vice versa to the negative bow.

**Figure 2:**
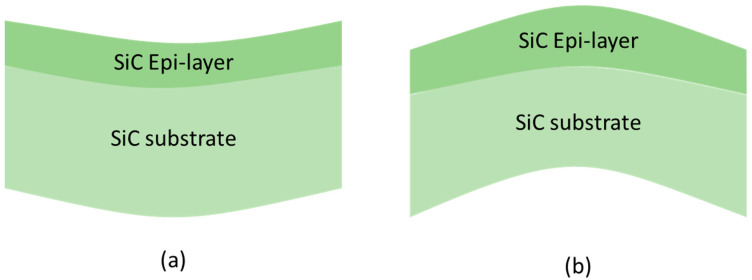
Schematic of wafer bowing: (**a**) negative bowing (**b**) positive bowing.

**Figure 3:**
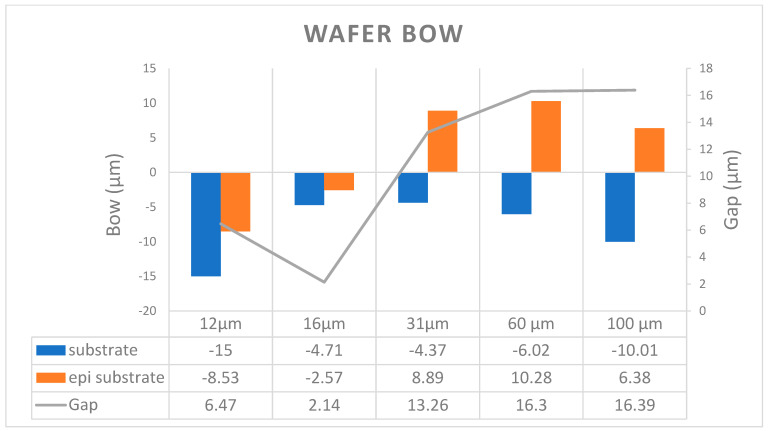
Relationship between SiC epi-layer thickness and wafer Bow.

The schematic in Figure 2 shows the wafer bowing phenomenon and Figure 3 illustrates the wafer bowing behavior from the substrate (blue bar) to different epitaxial layer thicknesses (orange bar). The gap between the substrate and the epitaxial wafer was calculated and plotted in the figures using a line chart (secondary axis). We observed that all the as-polished wafers were negative bowing, indicate a tensile stress distributed with Si-face toward up. But it shifts toward the positive when a 12 μm SiC epitaxial layer growth on the wafer, with a significant increase occurring when the epitaxial layer thickness exceeds 31 μm. Nadeemullah A. Mahadik et al. has reported wafer shape change may come from lattice plane curvature, and it can arise from doping variation, thermal stress, and extended defect concentrations [16]. Meanwhile, Fumihiro Fujie et al. found wafer warp increased with thicker epi layer growth and the influence of the difference in the lattice constants caused by the difference in nitrogen concentrations between the n^−^ epilayer and the n^+^ substrate; the stress distribution was calculated by finite element method [8]. In this section, our research work regarding wafer bowing phenomenon has positive correlation with the previous research work. From the observation, it is essential to further study stress compensation by increasing substrate thickness or generate more tensile stress during substrate wafering process.

### 3.2. Wafer TTV and LTV Behavior with Epi Layer Thickness

Unlike the wafer bow measurement methods, wafer TTV and LTV were measured under a clamped wafer status and calculated by summing the maximum and minimum gaps between the wafer surface and the focal plane. TTV indicates the total thickness variation in a wafer, while LTV indicates site thickness variation in a specific local area. In our study, LTV data were collected from the maximum value of 148 squares, each 10 mm × 10 mm on the wafer.

**Figure 4:**
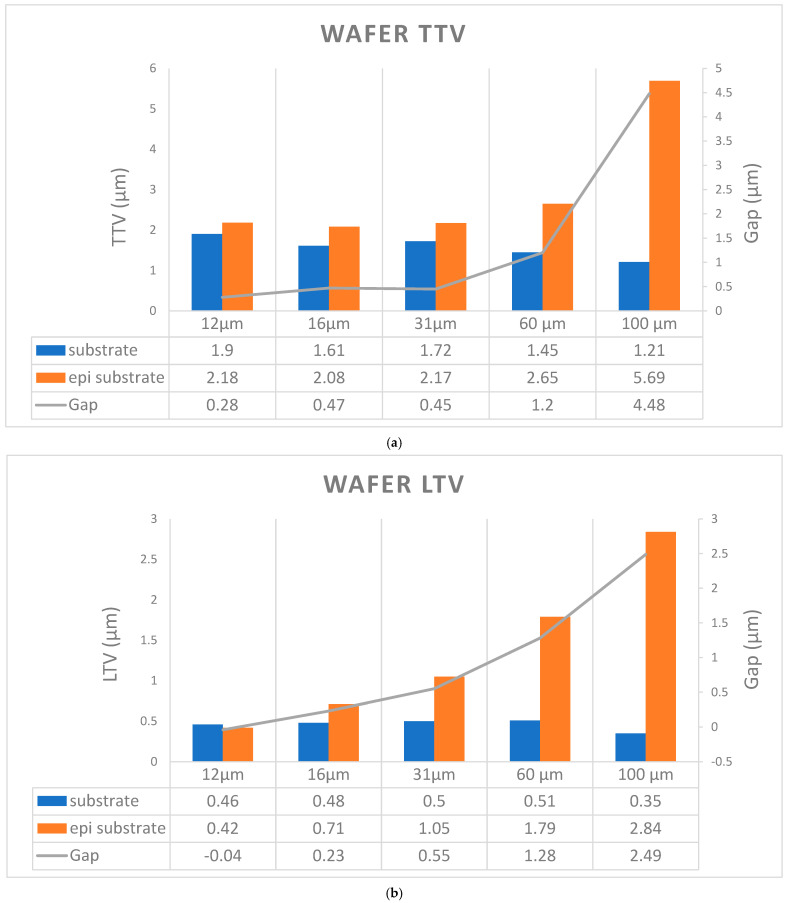
Relationship between SiC epi-layer thickness and wafer (**a**) TTV and (**b**) LTV.

Figure 4a,b show that TTV and LTV have a positive correlation with epitaxial layer thickness. We also observed that the slope in the line chart of both TTV and LTV gaps increased significantly after a 31 μm epitaxial layer thickness. From the LTV mapping diagrams, we observed that the maximum LTV value consistently occurs in the wafer edge area.

**Figure 5:**
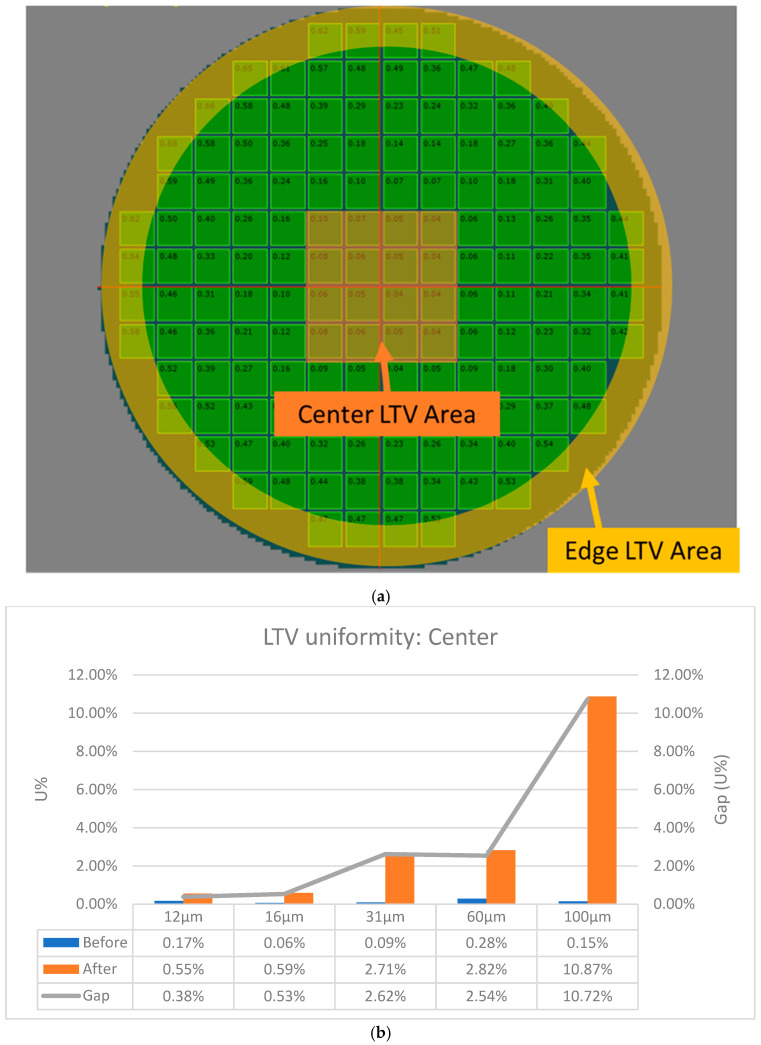

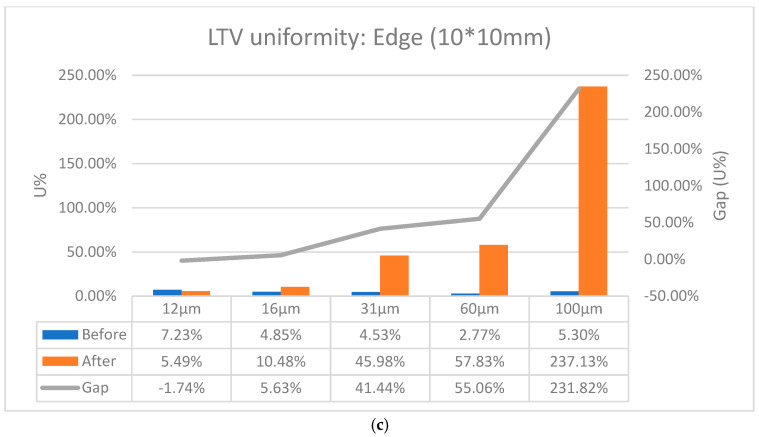
Relationship between SiC epi-layer thickness and wafer center/edge LTV uniformity. (**a**) schematic diagram of wafer center and edge LTV sampling points; (**b**) wafer center area LTV uniformity varied by epi layer thickness; (**c**) wafer edge area LTV uniformity varied by epi layer thickness.

To further understand the wafer LTV behavior at each epitaxial layer thickness, we calculated the uniformity variation in the wafer center area and edge area separately, as shown in Figure 5a. The formulation for LTV uniformity is shown in Equation (1):

(1) $$LTV\;Uniformity\;\%=\frac{Max.LTV-Min.LTV}{2*Average\;(LTV)}$$

The relationships between wafer center and edge area LTV uniformity and epitaxial layer thickness are shown in Figure 5b,c. Three groups of LTV uniformity gaps can be observed: Group 1 with 12 μm and 16 μm epitaxial thickness, Group 2 with 31 μm and 60 μm epitaxial thickness, and Group 3 with 100 μm epitaxial thickness. In Group 1, wafer edge LTV uniformity is about 10 times higher than that of the wafer center. This increases to 18.7 times in Group 2 and 21.6 times in Group 3. From the analysis data in Section 3.1 and Section 3.2, even the epitaxial layer uniformity can be controlled in 1.0–1.5%, but we observed wafer deformation increase with epi-layer thickness increased. The results of this section highlight the importance of future research on the investigation methods for controlling wafer bow and LTV uniformity as the industry moves toward thicker epitaxial layer growth for high-voltage power device manufacturing.

### 3.3. As-CMP Wafer Scratch Behavior with Epi Layer Thickness

Table 1 and Table 2 present a comparative analysis of defects observed in SiC substrates and epitaxial wafers, respectively. Previous studies have reported that certain substrate defects can extend into the epitaxial layer, leading to the formation of new defect types that negatively impact the electrical performance of SiC-based devices. However, there remains a lack of comprehensive investigation into the transfer of chemical mechanical planarization (CMP) induced scratches from the substrate to epitaxial layers of varying thickness.

Our observations reveal a notable reduction in visible scratches on the SiC substrate following the epitaxial growth process. As shown in Table 3, a comparison of defect maps indicates that scratch locations on the substrates do not consistently correlate with those observed on the corresponding epitaxial wafers, suggesting effective mitigation of CMP induced surface damage during epitaxial processing.

**Figure 6:**
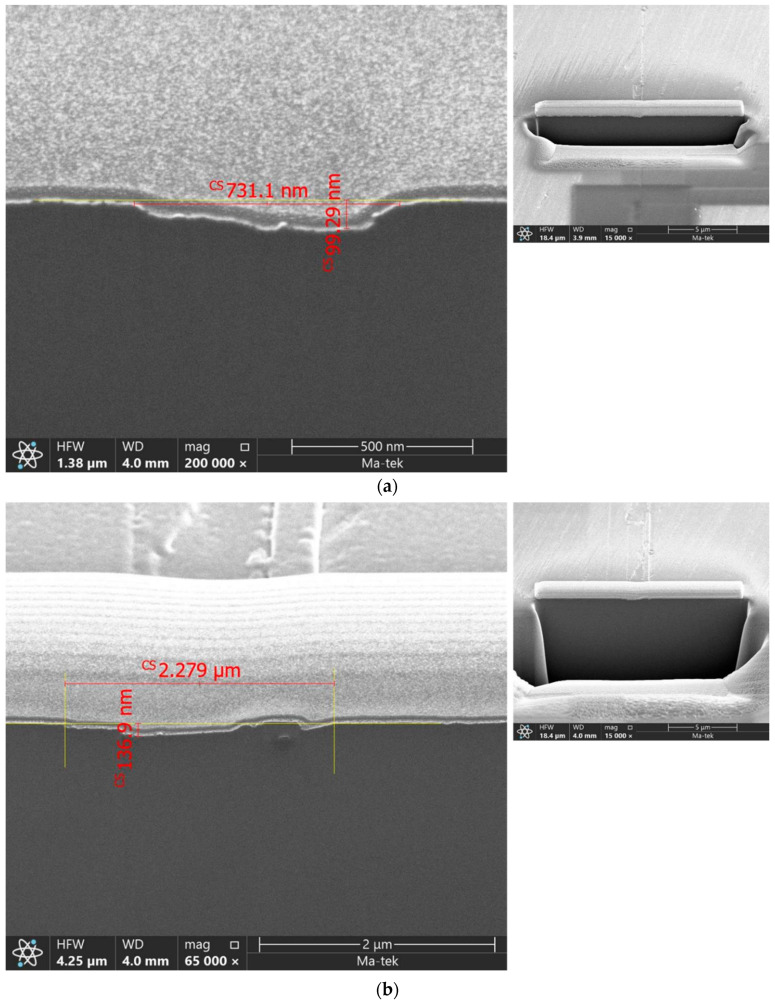
SEM and FIB inspection from 2 different scratches on the same SiC substrate after CMP process: (**a**) scratch depth of 99.29 nm and (**b**) scratch depth of 136.9 nm.

Table 4 further supports this conclusion with data from two additional prime grade SiC substrates, each used for the growth of 12 µm-thick epitaxial layers. One substrate exhibited a relatively high density of surface scratches, while the other showed minimal surface damage post-CMP. Scanning electron microscopy (SEM) and focused ion beam (FIB) analysis (Figure 6) measured scratch depths of 99.29 nm and 136.9 nm, respectively, prior to epitaxy.

**Figure 7:**
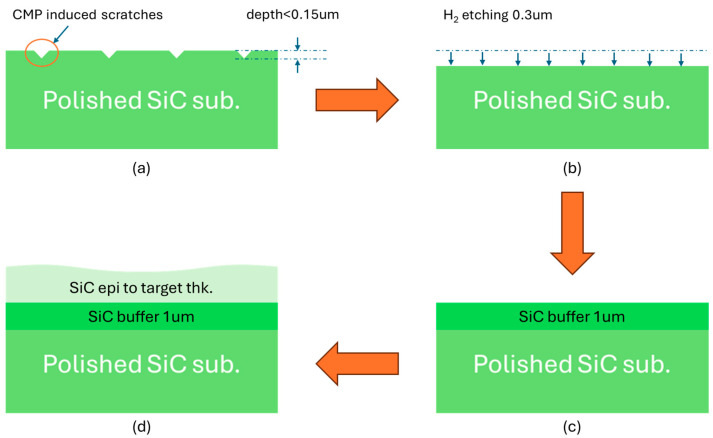
Schematic of CMP induced scratch removal in the epitaxial process steps (**a**) as-polished SiC wafer and (**b**) surface etching by H_2_ treatment in the process step 2 (**c**) 1μm buffer layer growth and in the process step 3 (**d**) epi layer growth to the target thickness.

Several studies have shown that substrate surface roughness and certain types of crystalline defects can be improved through hydrogen (H_2_) etching treatments [17,18,19]. Building upon the research motivation outlined in the introduction, our findings help explain why CMP-induced scratches can be tolerated within industrial specifications. As illustrated in Figure 7, the defect mitigation mechanism involves multiple stages: CMP-induced scratches present on the substrate surface (Figure 7a) are substantially etched away during H_2_ treatment (Figure 7b), with etching depths exceeding twice the measured scratch depth. A subsequently grown buffer layer (Figure 7c) further suppresses defect propagation, thereby reducing the likelihood of CMP-induced scratches extending into the main epitaxial layer (Figure 7d).

Our previous study [20] demonstrated the potential for substrate crystal defects to propagate into or transform within the epitaxial layer. The current study extends this understanding by showing that CMP-induced surface scratches can be effectively mitigated through optimized epitaxial growth processes, thereby minimizing their impact on epitaxial layer quality.

## 4. Conclusions

This study investigates the impact of epitaxial layer thickness on SiC wafer geometry, total thickness variation (TTV), local thickness variation (LTV), and the propagation of CMP-induced scratches into the epitaxial layer.

Wafer flatness measurements indicate a shift from negative bowing in as-polished substrates to positive bowing as the epitaxial layer thickness increases, with a significant transition occurring beyond 31 μm. The findings align with prior research attributing wafer shape changes to factors such as lattice curvature, doping variations, and thermal stress. Given the observed correlation, further studies on stress compensation strategies—such as increasing substrate thickness or modifying wafering processes—are crucial for controlling wafer bow in high-voltage SiC device manufacturing.

TTV and LTV analysis revealed a direct relationship between epitaxial layer thickness and thickness variation, with the most substantial increases observed beyond 31 μm. LTV mapping demonstrated that edge regions consistently exhibit the highest variations, with uniformity gaps widening significantly as epitaxial thickness increases. This highlights the need for improved process control strategies to maintain wafer geometry integrity in thick-epi SiC wafers.

Regarding CMP-induced scratch propagation, defect mapping, and SEM/FIB imaging showed that substrate scratches of around 100 nm depth do not transfer directly into the epitaxial layer. Although CMP introduces surface scratches, these are largely etched away during the hydrogen pre-treatment step and further suppressed by the buffer layer, leading to minimal transfer of substrate scratches into the epitaxial layer.

Overall, this work contributes to the understanding of substrate-to-epitaxial process integration, addressing critical challenges in SiC power device fabrication and supporting the industry’s transition to thicker epitaxial layers for next-generation high-power electronics.

## Figures and Tables

**Table 1:** SiC substrate defect inspection data.

Defect_Substrate	12 μm	16 μm	31 μm	60 μm	100 μm
Scratch	5	2	4	16	28
Particle	4	1	7	6	3
Pit	0	0	0	0	0
Stain	0	0	0	0	0
Macro Pit	0	0	1	0	1
Micro Pipe	0	0	0	0	0
IDL	0	5	13	29	44
Vis Stacking Fault	0	1	1	0	0
Small Particle	130	148	103	165	166
Bump	0	1	4	1	0
Total Defect count	139	158	133	217	242

**Table 2:** SiC epi wafer defect inspection data.

Defect_Epi Wafer	12 μm	16 μm	31 μm	60 μm	100 μm
Scratch	0	0	0	1	2
Particle	33	11	0	0	1
Shallow Triangle	28	26	48	21	26
Pit Triangle	24	14	24	7	40
BPD	13	7	2	4	17
Carrot	2	1	3	0	2
BSF	0	0	1	1	9
Large Particle	9	14	2	2	21
Triangle	5	4	6	1	1
Downfall	0	3	5	1	0
SSF	56	135	91	1	3
Total Defect count	170	215	182	39	122

**Table 3:** Defect map of substrate and different epi layer thickness.

	Substrate	Epi Wafer
12 μm	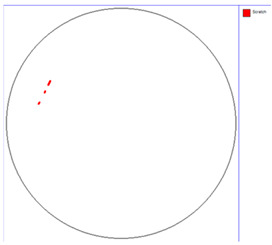	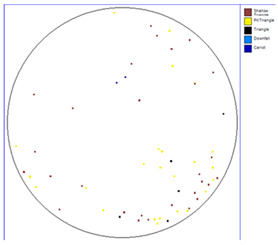
16 μm	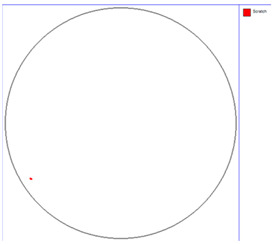	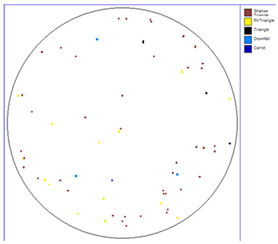
31 μm	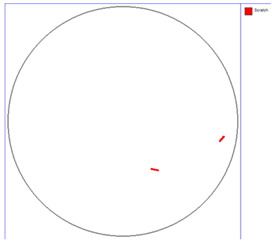	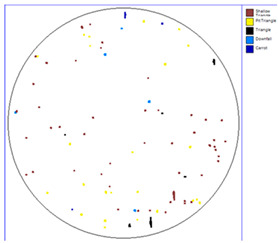
60 μm	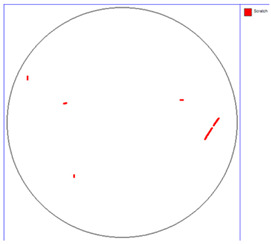	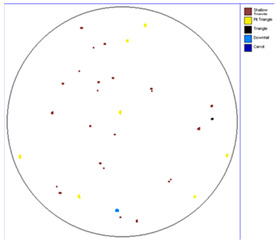
100 μm	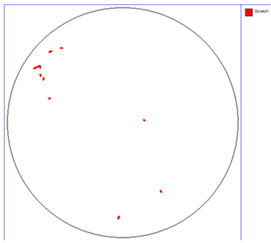	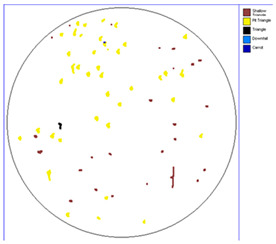

**Table 4:** Defect map of substate and epi wafer with different initial scratches levels.

	Substrate	Epi Wafer (12 μm)
Higher scratches density substrate	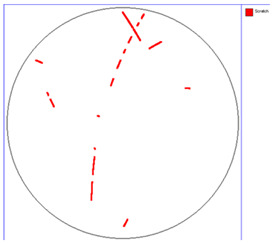	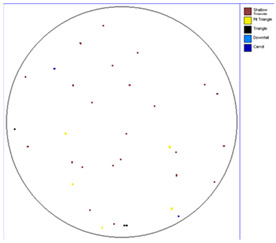
Lower scratches density substrate	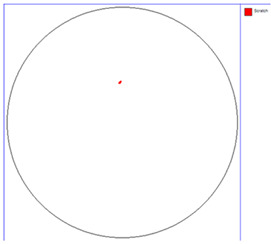	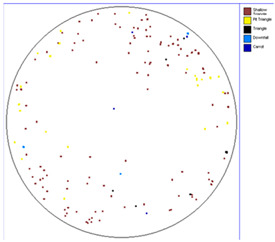

## Data Availability

The original contributions presented in this study are included in the article. Further inquiries can be directed to the corresponding authors.
